# Complete Genome Sequence of the Lytic Giant Bacteriophage pT24 Infecting *Tenacibaculum* spp., Isolated from a Shrimp Culture Pond

**DOI:** 10.1128/genomeA.00081-17

**Published:** 2017-07-06

**Authors:** Ho Viet Khoa, Yuki Midorikawa, Tsubasa Uchino, Toshihiro Nakai, Goshi Kato, Hidehiro Kondo, Ikuo Hirono, Matthura Labaiden, Sataporn Direkbusarakom, Motohiko Sano

**Affiliations:** a School of Marine Science, Tokyo University of Marine Science and Technology, Tokyo, Japan; b Graduate School of Biosphere Science, Hiroshima University, Hiroshima, Japan; c School of Agricultural Technology, Walailak University, Nakhon Si Thammarat, Thailand

## Abstract

The lytic bacteriophage pT24, which infects *Tenacibaculum* spp., was isolated from the water of a whiteleg shrimp (*Litopenaeus vannamei*) culture pond in Thailand. This giant bacteriophage with myovirus morphology comprised 234,670 bp with 296 predicted genes.

## GENOME ANNOUNCEMENT

The genus *Tenacibaculum* is a member of the family *Flavobacteriaceae* and is an abundant and important component of marine bacterial ecosystems (1–3). We isolated *Tenacibaculum* sp. T24—a Gram-negative, aerobic, gliding motile, long-rod bacterium that produces yellow pigment—from the water of a whiteleg shrimp (*Litopenaeus vannamei*) culture pond in Thailand. The lytic bacteriophage pT24 with myovirus morphology was isolated from the water of a shrimp-rearing pond in Thailand by screening on T24. Here, we report the complete genome sequence of phage pT24, which can infect the species *T. mesophilum* and *T. discolor*.

DNA of the phage was sequenced with the Illumina MiSeq platform using the Nextera XT library preparation kit and the MiSeq version 3 reagent kit (600 cycles). The sequence reads were imported into CLC Genomics Workbench version 9 (CLC bio) and *de novo* assembled. The obtained linear DNA sequences were confirmed by PCR, with a primer set designed at both ends of the sequence, followed by Sanger sequencing. The coding sequences (CDSs) were predicted by GeneMarkS (4), followed by annotation using Blast2GO (5); tRNAs were found using tRNAscan-SE version 2.0 (6).

In total, 1,182,866 reads were generated (1,290× coverage of the genome), and their average length was 255.84 bp. Phage pT24 had 234,670 bp of linear genomic DNA with a GC content of 28.85% and 4 tRNAs identified. A total of 296 CDSs were predicted, and among the 41 annotated genes were those encoding for DNA ligase, DNA helicase, DNA polymerase, large terminase protein, ribonucleotide reductase, DNA topoisomerase, RNA ligase, and thymidylate synthase. Twenty of the 41 annotated proteins revealed similarities to those of the “giant phage” *Sphingomonas* phage PAU (GenBank accession no. NC_019521) (7) at 42.9 to 59.7% identity.

This is the first report of a *Tenacibaculum* phage genome sequence. This newly sequenced genome information will be useful for studies on the dynamics of phage-infecting environmental bacteria in shrimp culture ponds and on the interactions between phages and their hosts, as well as to clarify the impact of lytic phages on the microbial community structure in the pond.

### Accession number(s).

The complete genome sequence of bacteriophage pT24 was submitted to DDBJ/GenBank under the accession number LC168164.
